# Interventions for improving pharmacist-led patient counselling in the community setting: a systematic review

**DOI:** 10.1186/s13643-018-0727-4

**Published:** 2018-05-02

**Authors:** Sinaa Al Aqeel, Norah Abanmy, Hiba AlShaya, Albatoul Almeshari

**Affiliations:** 1 Clinical Pharmacy Department, College of Pharmacy, King Saud University, Riyadh, Kingdom of Saudi Arabia; 2 Pharmaceutical Care Services, King Abdulaziz Medical City, National Guard Health Affairs, PO Box 376316, Riyadh, 11335 Kingdom of Saudi Arabia

**Keywords:** Community pharmacy, Community pharmacy services, Pharmacies, Professional practice, Patient education, Counselling

## Abstract

**Background:**

Pharmacist counselling is an important service that has been associated with improved outcomes. The primary aim of this review was to identify, describe, and determine the effectiveness of interventions for improving the counselling practice of community pharmacists.

**Methods:**

We searched PubMed (from January 1990 to June 2017) and the Cochrane Library (June 2017). To supplement our database searches, we searched Google Scholar for papers that cited the identified studies. We included only studies that reported the impact of the intervention on pharmacists’ behaviour during counselling. We searched for data from studies with randomised trials, non-randomised trials, controlled before-after studies, or interrupted time series study designs. Parameters including selection bias, performance bias, detection bias, and attrition bias were assessed. The data were narratively synthesised.

**Results:**

We screened 2335 abstracts and 59 full-text articles and included 17 RCTs. Overall, three studies were determined to have a high risk of bias, and 14 studies were determined to have an unclear risk of bias. Fifteen studies investigated multifaceted interventions that included two or more components. The most commonly used interventions were educational meetings (*n* = 14), educational materials (*n* = 9), educational outreach visits (*n* = 5), feedback (*n* = 5), guidelines (*n* = 5), and local opinion leaders (*n* = 2). Outcomes were measured using simulated patient visits (*n* = 10), and the self-reported outcomes of patient or pharmacists (*n* = 6). Most of the included studies (*n* = 11) reported some degree of improvement in counselling practices.

**Conclusions:**

The included studies showed that educational meetings combined with educational materials, outreach visits, and feedback can improve pharmacist counselling in community settings. However, the unclear risk of bias and poor quality of reporting intervention components necessitate caution in interpreting the findings. Recommendations for future studies based on the evidence gap identified in this review are presented.

**Electronic supplementary material:**

The online version of this article (10.1186/s13643-018-0727-4) contains supplementary material, which is available to authorized users.

## Background

According to the Joint International Pharmaceutical Federation (FIP)/World Health Organization (WHO) guidelines for good pharmacy practice, the mission of pharmacy practice is to “contribute to health improvement and to help patients with health problems to make the best use of their medicines” [1]. Pharmacist-led counselling is an important service that has been associated with improved clinical outcomes, quality of life, drug/disease knowledge, satisfaction, and reduced health service utilisation among patients [2].

There is no accepted definition of counselling. According to Puspitasari et al. [3], researchers either operationally define counselling or refer to specific counselling guidelines in the literature. Pharmacy researchers operationally define counselling as giving advice or providing information on medications, while others focus more on the goal of counselling, i.e. ensuring that patients understand the optimal use of medications to improve their quality of life [3]. In a review focusing on the conceptualisation and measurement of pharmacist-patient communication, Shah and Chewning [4] discuss the differences in counselling definitions between the professional counselling literature and other published pharmacy literature. The professional counselling literature, represented by an international interdisciplinary journal, defines counselling as an “individualised process involving guidance and collaborative problem solving to help the patient better manage their health problems” [5], while other published pharmacy literature uses the term “counselling” to refer to the provision of information regarding how to take the drug product properly [4]. Furthermore, the terms “communication”, “counselling”, “education”, and “information provision” have been used interchangeably in the literature, disregarding subtle differences in their meaning [4]. Patient education, for example, is defined as “a planned learning experience using a combination of methods such as teaching, counselling, and behaviour modification techniques that influence patients knowledge and behaviour” [5]. Thus, according to this definition, counselling is an aspect of patient education [4].

In addition to dispensing prescription and non-prescription medications, community pharmacists have great potential to be the first contact for patients seeking treatment for minor ailments. Community pharmacists also have an increasing role in public health through the promotion of healthy lifestyles. Pharmacist-patient interactions in the community setting may also address diet, device use, exercise, referrals, or other non-medication issues. The community setting offers many advantages, such as long opening hours, accessibility, and familiarity.

Studies investigating the counselling practices of community pharmacists have indicated that their elicitation of information prior to supplying medicine, detection of drug interactions, and counselling content are of suboptimal quality [6–11]. Counselling in a community setting is a complex process [12], which may explain the poor quality of community counselling practices.

Previous reviews have examined the impact of community pharmacy services, such as counselling, on patient outcomes [2, 13, 14], quality of counselling [10], verbal counselling rates [3], types of information provided during counselling [3], and the conceptualisations, definitions, and measurements of pharmacist-patient communication in the community setting [4]. Few studies, however, have examined interventions to improve community pharmacy services. Patwardhan et al. [15] reviewed literature published up to 2010 on interventions for enhancing community pharmacists’ cognitive services, defined as professional services provided by pharmacists to a patient that are either judgemental or educational in nature. Watkins [16] searched six databases up to 2014 for literature on implementation strategies for clinical guidelines to community pharmacy and their impact on the quality of care provided by community pharmacists, such as adherence to recommended practices or guidelines. In December 2017, Seubert et al. [17] published a review of literature published between 2000 and 2017 on interventions aimed at improving communication between consumers and pharmacy personnel during consultations for medicines that are provided without a prescription.

These reviews focused on general cognitive services rather than on counselling specifically; on specific interventions, such as guidelines; or on specific groups of medications. To date, however, no systematic reviews have focused on interventions for improving counselling in different situations, including prescription and non-prescription medications, consultations for minor ailments, and health promotion. Given the suboptimal quality of counselling noted earlier, we need to close the gap in knowledge regarding which interventions might lead to optimal counselling in the community setting.

The primary aim of this review was to identify, describe, and determine the effectiveness of interventions for improving the counselling practice of community pharmacists. The secondary aim was to provide recommendations for future studies due to the evidence gap identified in this review. In this review, “counselling” is used as a broad umbrella term that encompasses all definitions outlined in the background section.

## Methods

### Study design

The review was guided by the recommendations of the Cochrane Handbook [18] and York Systematic Review Centre for Reviews and Disseminations (CRD) guidelines [19]. The reporting of the review complied with the Preferred Reporting Items for Systematic Reviews and Meta-Analyses (PRISMA) statement [20]. The protocol was not registered on PROSPERO.

### Search methods

We searched PubMed from January 1990 to June 2017. We limited our search to fully published articles written in the English language. The time and language restrictions were due to resource limitations. The Cochrane Library was also searched, including the Cochrane Central Register of Controlled Trials (CENTRAL), the Health Technology Assessment Database, the National Health Service (NHS) Economic Evaluation Database, and the Cochrane Group’s specialised register (including the Cochrane Effective Practice and Organisation of Care (EPOC) group register).

The search strategies comprised the following three key concepts: study design, community pharmacy setting, and counselling. The search strategies were designed specifically for each concept and were guided by similar previous studies [2, 3, 13] and combined. The terms used to search the databases are listed in Table 1. The search was conducted in June 2017. To supplement the results of the online searches, we searched for articles that cited the identified studies using Google Scholar and screened these articles for potential studies. We also screened the bibliographies of the identified studies. We did not search for grey literature, such as conference abstracts and reports, as the lack of details regarding study design would not allow for risk of bias and quality assessments.

**Table 1:** Search strategy

Database	Search terms	Hits
PubMed	Counseling"[Mesh] OR Counseling OR Counselling OR Counsel* OR "Patient Education as Topic"[Mesh]) OR Patient Education) OR consult*OR interact* OR advi* AND Community pharmacy OR "Community Pharmacy Services"[Mesh] OR independent pharmacies OR retail pharmacy OR retail pharmacies OR chain pharmacy OR chain pharmacies) AND Pharmacy[MeSH Major Topic]) OR "Pharmacy"[Mesh] OR "Pharmacies"[Mesh]) OR pharmacists OR "Pharmacists"[Mesh] OR pharmacies AND Randomized Controlled Trial OR Controlled Clinical Trial OR Comparative Study OR intervention studies OR time adj series OR pre test OR pretest OR posttest OR post test) OR impact OR chang* OR evaluat* OR intervention OR random allocation OR evaluation studies Filters: Publication date from 1990/01/01 to 2017/06/07; English	979
Cochrane Library	Counseling or Counselling or Counsel or Advice or Education AND community Pharmacist or community Pharmacists or community Pharmacy or community Pharmacies	638
Cochrane Database of Systematic Reviews		200
Cochrane Central Register of Controlled Trials		415
Health Technology Assessment Database		1
NHS Economic Evaluation Database		22

### Study selection

#### Types of participants

Studies were eligible for inclusion if they included pharmacists who delivered services in a community pharmacy. The community pharmacy was defined as a pharmacy in the community that is accessible to all and not based in a hospital or clinic or online [5]. As the review question focus on pharmacists, we excluded studies solely targeting pharmacy technicians and studies assessing pharmacy students’ counselling skills.

#### Types of interventions

We included all types of interventions intended to improve pharmacist-led counselling in a community pharmacy setting. The review aim was to identify the types of interventions examined in the literature; therefore, we imposed no restrictions on the characteristics of interventions, such as mode of delivery (e.g. face-to-face, online), format, frequency, or length.

#### Type of comparison

Control groups should have received no intervention, a different intervention, or “usual care” as defined by the individual study’s authors.

#### Types of outcomes

We included only studies that measured the impact of the intervention on the pharmacists’ behaviour during counselling, such as asking questions, providing information, or dispensing appropriate medication. Therefore, we excluded studies that measured pharmacists’ satisfaction, attitudes, or theoretical knowledge after the intervention. We also excluded studies that measured only the feasibility or acceptance of an intervention without reporting the impact of the intervention on counselling practices. Studies that examined the impact of counselling on patient outcomes only and did not report outcomes related to the pharmacists’ counselling practice were excluded. One such example is a study that reported the impact of a hypertension management programme delivered by community pharmacists on hypertension control, adherence to prescribed regimens, and quality of life.

#### Types of studies

We searched for data from studies with randomised trials, non-randomised trials, controlled before-after studies, or interrupted time series study designs as defined in the Cochrane Effective Practice and Organisation of Care (EPOC) guidelines [21]. We did not include other study designs because they provide little, if any, reliable evidence of the effects of interventions.

### Data collection

Two authors independently screened the titles and abstracts of identified citations for potential eligibility using a standardised screening guide. Full articles were retrieved for all studies that appeared to be eligible for inclusion in the review and any studies for which it was not possible to draw firm conclusions regarding eligibility based on the abstract alone. Then, two authors (SA and NA) read the full texts of the potential papers to confirm whether they satisfied the inclusion criteria. Disagreement was solved by consensus.

The data extraction was performed by a single author (SA) using a data-extraction instrument that encompassed the author, year of the study, setting, participants, sample size, intervention assessed, outcome measures, and main findings. The interventions were grouped according to the EPOC taxonomy of interventions [21], which includes the following four main domains of interventions: delivery arrangements, financial arrangements, governance arrangements, and implementation strategies. Other authors (NA, HA, AA) reviewed the extracted data.

### Risk of bias

The risk of bias for all included studies was independently assessed using the domains suggested by EPOC [21] to assess the risk of bias: random sequence generation, allocation concealment, incomplete outcome data, knowledge of the allocated interventions adequately prevented during the study, protection against contamination, selective outcome reporting, similar baseline outcome measurements, and similar baseline characteristics, and other outcomes. Then, we summarised the assessments of the risk of bias across domains for each study. An overall rating of low risk of bias was assigned if a low risk of bias was scored for all key domains. An overall rating of high risk of bias was assigned if a high risk of bias was scored for one or more key domains. An overall rating of unclear risk of bias was assigned if an unclear risk of bias was scored for one or more key domains.

### Data synthesis

The data were narratively synthesised due to the profound methodological heterogeneity. The guidelines for narrative synthesis in systematic reviews provided by the York Systematic Review Centre for Reviews and Disseminations (CRD) were followed [19].

## Results

### Study selection

**Fig. 1:**
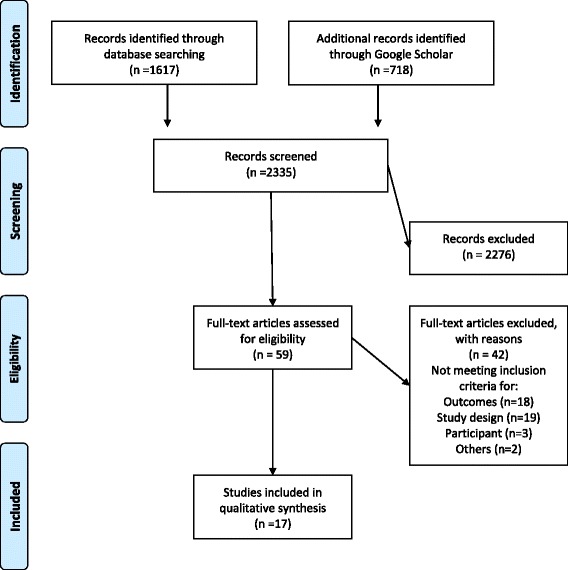
Flow diagram of study selection

The PRISMA flow diagram of study inclusion and the PRISMA checklist are provided in Fig. 1 and Additional file 1, respectively. The initial search yielded 2335 citations. Based on the abstracts and titles, 2276 papers were excluded. The remaining 59 articles were retrieved in full text and reviewed, and 42 articles were excluded. Additional file 2 lists articles and reason for exclusions. In total, 17 papers met the inclusion criteria and were reviewed for this paper [22–38]. No additional references were identified by searching the bibliographies.

### Characteristics of the included studies

The characteristics of the included studies are described in Table 2. All included studies used randomised trial designs. The unit of randomisation was either pharmacists, pharmacies, or districts. In total, 16 studies included in this review were at least 10 years old. Four studies were conducted in Australia, four studies were conducted in the USA, two studies each were conducted in Canada, Scotland, Peru, and Vietnam, and one study was conducted in Switzerland.

**Table 2:** Characteristics of included studies

Study (year), country	Study design	Sample size	Retention rate	Targeted care type	Intervention category^a^	Outcome measures	Outcome assessment	Follow-up
Basheti (2009), Australia	RCT	IG = 16 CG = 15	6 months: IG 15/15 CG 12/16 (87%) 2 years: 12/16 9/15 (67%)	Asthma inhalers	1, 2, 4	Pharmacists’ technique demonstration skills	A researcher assessed pharmacists using a scale of 0–9	3 and 6 months and 2 years after training
Chalker (2005), Vietnam and Thailand	Cluster RCT	Hanoi IG = 34 CG = 34 Bangkok IG = 39 CG = 39	Hanoi 28/34 27/34 (81%) Bangkok IG 34/39 CG 35/39 (91%)	Antibiotic/oral steroids	1, 2, 3, 5	Illegal dispensing of prescription and asking questions and giving advice	Simulated patient visits	3 months after each intervention
Chuc (2002), Vietnam	Cluster RCT	IG = 34 CG = 34	IG 29/34 CG 29/34 (85%)	Antibiotic/oral steroids/STD/ARI	2, 3, 5, 6	ARI: not dispensing antibiotics and asking about breathing STD: advice to go to the doctor and dispensing the correct treatment Antibiotic and steroids: prescription request	Simulated patient visits	1 month after each intervention
de Almeida Neto (2000), Australia (1)	RCT	IG = 15 CG = 15	IG13/15 CG14/15 (90%)	Non-prescription analgesics	1, 4, 6	Observations on 11 pharmacists behaviour measures such as the use of open-ended questions	Audiotaped simulated patient visits	6 weeks
de Almeida Neto (2000), Australia (2)	RCT	IG = 16 CG = 8	IG 14/16 CG 8/8 (92%)	Non-prescription analgesic	1, 4, 6	Observations on 9 pharmacists behaviour measures such as asking if the if the consumer had used the medication before	Simulated patient visits	14 weeks
Dolovich (2007), Canada	RCT	IG = 33 CG = 31	IG 29/33 CG 30/31 (92%)	Asthma treatment	1, 2	The number of pharmacists-facilitated plans Pharmacists’ general communication skills using the Global Rating Scale	Simulated patient visits	3–5 weeks
Garcia (1998), Peru	Cluster RCT	IG = 90 CG = 90	IG 86/90 CG 88/90 (97%)	Sexually transmitted diseases	1, 2	Symptoms recognition, offering of recommended treatment, patient referral and education and counselling frequency	Simulated patient visits	2–3 months
Garcia (2003), Peru	Cluster RCT	IG = 884; 750 finished training CG = 883	IG 100 and CG 100 from each group were assessed	Sexually transmitted diseases	1, 2, 3	Symptoms recognition, offering of adequate management, recommend use of condoms, recommend treatment of partner, patient referral and education and counselling frequency	Simulated patient visits	1, 3, and 6 months
Kimberlin (1993), USA	RCT	IG = 57 CG = 45 (762 elderly patients)	(100%)	Drug-related problems in elderly	1, 2	Drug use variable reports of pharmacist patient care activities, patients’ knowledge about the drug, adherence, and drug therapy problems	Patient-reported assessment	1 and 3 months
Lalonde (2008), Canada	Cluster RCT	IG = 22 CG = 20	IG 14/22 CG 15/20 (69%) 88/102 (86%) pharmacists	Drug-related problems in kidney disease patient	1, 7	The numbers of pharmacists’ written recommendations to physicians (pharmaceutical opinions), refusals to dispense a medication, the number and description of requests to the consultation service, and pharmacists’ satisfaction with the programme	The community pharmacy dispensing chart and satisfaction questionnaire	6 months
Mayer (1998), USA	Cluster RCT	IG = 27 CG = 27	IG = 27/27 CG = 27/27 (100%)	Skin cancer	2, 4	The rate of skin cancer prevention counselling	Simulated patient visits	3 weeks
Patwardhan (2012), USA	Cluster RCT	IG = 8 CG = 8	IG 8/8 CG 8/8 (100%)	Smoking cessation	1, 2, 3	Number of customers asked about tobacco use, number of tobacco users advised to quit, number of users enrolled in the quit line via Fax to Quit (active referral), and number of quit line cards given (passive referral). Pharmacists self-efficacy was also measured	The quit line’s Fax to Quit reports and the pharmacists self-report	1 month
Prokhorov (2010), USA	Cluster RCT	IG = 45 CG = 38	IG 39/45 CG 36/38 (90%)	Smoking cessation	1	Pharmacists counselling activities for each of the 5 A’s counselling practice model: ask, advice, assess, assist, and arrange. Pharmacists’ perceived ability, confidence, and intention (ACI) to address counselling activity	Patient-reported assessments and pharmacists self-report	12 months
Reeves (2007), Australia	Cluster RCT	IG = 31 CG = 21	IG 31/31 CG 21/21 (100%)	Aspirin in eligible patients with diabetes	6, 8	The rate of clinical interventions	Pharmacists electronic documentation	6 weeks + weeks post-intervention
Sigrist (2002), Switzerland	RCT	IG = 14 CG = 13	IG 14/14 CG 13/14 (100%)	Non-prescription analgesics	1, 4	Improvement on 15 attributes related to non-prescription services	Audiotaped simulated patient visits	2 months
Sinclair (1998), Scotland	Cluster RCT	IG = 31 CG = 31	IG 31/31 CG 29/31 (97%)	Smoking cessation	1	The perceptions of customers and pharmacy personnel of the pharmacy support and self-reported smoking cessation rates	Patient-reported assessments	1, 4, and 9 months
Watson (2002), Scotland	Cluster RCT	IG 1 = 15 IG 2 = 15 IG 3 = 15 CG = 15	IG 1 15/15 IG 2 15/15 IG 3 15/15 CG 15/15 (100%)	*Vulvovaginal candidiasis*	1, 3, 6	The appropriate sale or non-sale of over the counter antifungal (based upon the guidelines)	Simulated patient visits	5–8 months

*RCT* randomised controlled trial, *IG* intervention group, *CG* control group

^a^*1* educational meetings: courses, workshops, conferences, or other educational meetings; *2* educational materials: distribution to individuals, or groups, of educational materials to support clinical care, i.e., any intervention in which knowledge is distributed; *3* educational outreach visits or academic detailing. Personal visits by a trained person to health workers in their own settings, to provide information with the aim of changing practice; *4* audit and feedback: a summary of health workers’ performance over a specified period of time, given to them in a written, electronic, or verbal format; *5* local opinion leaders; *6* clinical practice guidelines: systematically developed statements to assist healthcare providers and patients to decide on appropriate health care for specific clinical circumstances; *7* communication between providers: systems or strategies for improving the communication between health care providers; and *8* reminders

Only seven studies reported the sample size calculations [22, 24, 27, 35–38]. Twelve studies achieved follow-up with ≥ 90% of the participants; three studies achieved follow-up with 80–90% of the participants; and in two studies, the follow-up was less than 80% (see Table 2).

### Risk of bias

The risk of bias is presented in Table 3. Overall, three studies were determined to have a high risk of bias, and 14 studies were determined to have an unclear risk of bias.

**Table 3:** Risk of bias of included studies

Study (year)	Was the allocation sequence adequately generated?	Was allocation adequately concealed?	Was the study adequately protected against contamination?	Was knowledge of the allocated interventions adequately prevented?	Were incomplete outcome data adequately addressed?	Was baseline outcome measurement similar?	Was baseline characteristics similar?	Was the study free from selective outcome reporting?	Overall risk^a^
1. Basheti (2009)	Low	Unclear	Unclear	Unclear	Low	Low	Low	Low	Unclear
2. Chalker (2005)	Unclear	Unclear	Low	Unclear	Low	Low	Unclear	Low	Unclear
3. Chuc (2002)	Low	Unclear	Low	Low	Low	Low	Unclear	Low	Unclear
4. de Almeida Neto (2000)	Unclear	Unclear	Unclear	Unclear	Low	Unclear	Unclear	Low	Unclear
5. de Almeida Neto (2000)	Unclear	Unclear	Unclear	Unclear	Low	Unclear	Unclear	Low	Unclear
6. Dolovich (2007)	Low	Unclear	Unclear	Low	Low	Unclear	Low	Low	Unclear
7. Garcia (1998)	Unclear	Unclear	Low	Low	Low	Unclear	Unclear	Low	Unclear
8. Garcia (2003)	Low	Unclear	Low	Low	Low	Unclear	Unclear	Low	Unclear
9. Kimberlin (1993)	Unclear	Low	Unclear	Unclear	Low	Unclear	Low	Low	Unclear
10. Lalonde (2008)	Low	Low	Low	High	Low	Low	High	Low	High
11. Mayer (1998)	Unclear	Unclear	Low	Low	Low	Low	Unclear	Low	Unclear
12. Patwardhan (2012)	Low	Low	Low	Unclear	Low	Low	Low	Low	Unclear
13. Prokhorov (2010)	Unclear	Unclear	Low	Unclear	Low	Unclear	Low	Low	Unclear
14. Reeves (2007)	Low	Unclear	Low	High	Low	Unclear	Low	Low	High
15. Sigrist (2002)	Unclear	Unclear	Unclear	Low	Low	Low	Unclear	Low	High
16. Sinclair (1998)	High	Unclear	Low	Low	Low	Unclear	Low	Low	Unclear
17. Watson (2002)	Low	Unclear	Low	Unclear	Low	Low	Low	Low	Unclear

^a^Key for overall assessment of bias within a study; if low of bias for all key domains (low of bias); if unclear risk of bias for one or more key domains (unclear risk of bias); if high of bias for one or more key domains (high of bias)

In 11 studies, allocation was by pharmacies, community, or district, and it is unlikely that the control group received the intervention; therefore, the risk of contamination was scored low. In seven studies, the authors explicitly stated that the main outcomes were assessed blindly; therefore, the likelihood of detection bias was low. In four studies, the outcomes were extracted from the participating pharmacists’ documentation and thus were judged as unblinded assessments with a high risk of detection bias. We assessed that missing outcome data were unlikely to be related to the true outcome (most commonly, closure of the pharmacy) and that the reasons for missing data were similar across groups. Therefore, we assessed the attrition bias risk as low. In 17 studies, we judged that there was no evidence that outcomes were selectively reported as all the relevant outcomes in the “Methods” section were reported in the “Results” section; hence, the risk of bias from selective outcome reporting was scored as low.

### Types of interventions

All the identified interventions were categorised as implementation strategies (i.e. interventions designed to cause changes in the actions of healthcare organisations, the behaviour of healthcare professionals, or the use of health services by healthcare recipients) [21]. We further grouped these interventions according to the EPOC taxonomy [21] into subcategories, such as educational meetings, educational materials, educational outreach visits, reminders, audits, and feedback. Table 4 presents a definition for each category. As shown in Table 2, 15 studies investigated multifaceted interventions that included two or more components. The most commonly used interventions were educational meetings (*n* = 14), educational materials (*n* = 9), educational outreach visits (*n* = 5), feedback (*n* = 5), guidelines (*n* = 5), and local opinion leaders (*n* = 2). In five studies, pharmacists were supplied with materials to give to patients, such as leaflets, brochures, posters, and prompts, such as sunscreen samples. Additional file 3 provides details on the interventions.

**Table 4:** Professional interventions as per Cochrane EPOC review group (adapted from reference [21])

Intervention	Description
Category: interventions targeted at healthcare workers	
Distribution of educational	Distribution to individuals, or groups, of educational materials to support clinical care, i.e., any intervention in which knowledge is distributed. For example, this may be facilitated by the Internet, learning critical appraisal skills; skills for electronic retrieval of information, diagnostic formulation; question formulation
Educational meetings	Courses, workshops, conferences, or other educational meetings
Educational outreach visits	Personal visits by a trained person to health workers in their own settings, to provide information with the aim of changing practice
Audit and feedback	Any summary of clinical performance of healthcare over a specified period of time. The summary may also have included recommendations for clinical action. The information may have been obtained from medical records, databases, or patient observations
Clinical practice guidelines	Clinical guidelines are systematically developed statements to assist healthcare providers and patients to decide on appropriate health care for specific clinical circumstances (US IOM)
Local opinion leaders	The identification and use of identifiable local opinion leaders to promote good clinical practice
Reminders	Manual or computerised interventions that prompt health workers to perform an action during a consultation with a patient, for example computer decision support systems
Coordination of care and management of care processes	
Communication between providers	Systems or strategies for improving the communication between health care providers, for example systems to improve immunisation coverage in LMIC

The format, frequency, and length of educational meetings were categorised according to previously used criteria [39]. The format was categorised as either didactic or interactive. The frequency was categorised as frequent (more than 10), moderate (5–10), infrequent (2–4), and one-time only. The length of the educational meeting was categorised as prolonged (5 days or more), moderate (2–4 days), brief (1 day), and very brief (less than 1 day). The majority of the educational meetings were a combination of didactic and interactive formats (*n* = 13), were conducted once (*n* = 13), and were very brief (*n* = 14; see additional file 3). Only six studies indicated the framework used to develop the interventions, including the stages of change model (*n* = 4) [25, 26, 36, 37], the principles of motivational interviewing (*n* = 2) [25, 26], cognitive-behavioural and multi-modal therapy (*n* = 1) [30], the social cognitive theory (*n* = 1) [33], and the health belief model [36]. One study described the creation of the intervention in a qualitative study [33]. The control groups received no intervention (*n* = 12) or educational meetings on a different topic (*n* = 2). One study compared the dissemination of an evidence-based guideline via educational meetings, outreach visits, educational and outreach visits, and by post (control) (see Additional file 3).

The costs of the intervention were reported by three studies [24, 29, 38]. The participants’ opinions of the intervention were measured in three studies [31, 35, 36].

### Outcome measurement

The effectiveness of the interventions was measured using a range of outcomes, including changes in pharmacists’ behaviour, such as the use of open-ended questions; changes in the number of recommendations made by the pharmacists; and changes in the number of customers who received the service (see Outcome measures in Table 2).

The change in outcomes was assessed mainly using simulated patient visits (*n* = 10; see Outcome assessment in Table 2). A simulated patient, also known as a standardised patient, pseudo-patient, or mystery shopper, is a patient actor who is trained to simulate pre-determined situations in the course of teaching or evaluation [40]. Self-reports of patient or pharmacists were included as outcomes in four studies. Pharmacists’ documentations were reported in three studies. No study reported on the validity and reliability of the tool used to measure outcomes.

The outcome assessments occurred a few weeks after the interventions in most of the identified studies (see Follow-up in Table 2).

### Effects of the interventions

Additional file 3 provides details on interventions and outcomes reported in the included studies. This section provides a narrative synthesis of the evidence. First, we conducted a preliminary synthesis of the findings of the included studies, grouped according to targeted care. Then, we explored the relationships among the characteristics of the individual studies, their reported findings, and the findings of different studies.

We grouped the interventions according to targeted care as follows: promoting healthy life styles (*n* = 6), dispensing non-prescription medications (*n* = 4), dispensing prescription medications (*n* = 2), drug-related problems (*n* = 2), treatment recommendations (*n* = 2), and use of medical devices (*n* = 1).

The promotion of healthy lifestyles group involved intervention-targeted pharmacist counselling concerning smoking cessation (*n* = 3), sexually transmitted diseases (*n* = 2), and skin cancer prevention (*n* = 1). The interventions involved educational meetings supplemented with educational materials, outreach visits, and feedback. For smoking cessation studies, patient-reported outcome measures demonstrated an improvement in two studies and no effect in one study. For sexually transmitted diseases [28, 29], simulated patients reported significantly better recognition and management in one study and mixed results in the other. Simulated patients reported significantly improved rates of skin cancer counselling [32] in the intervention group (which received educational videos and onsite feedback on counselling performance) compared with the control group.

The dispensing of non-prescription medication group involved three studies on analgesics [25, 26, 36] and one study on antifungals [38]. Simulated patient visits were used to assess outcomes in all the studies. The interventions, which included educational meetings, onsite feedback, and protocol, improved significantly the process of non-prescription analgesics counselling. There was no difference in outcomes between the dissemination of an evidence-based guideline for the sale of antifungals by post, educational outreach visits, education meetings, or educational meetings combined with outreach visits.

The studies (*n* = 2) on dispensing prescription medications involved medications such as antibiotics and oral steroids and were conducted in three study sites [23, 24]. The multifaceted interventions involved educational meetings, educational materials, outreach visits, local opinion leaders, and guidelines. Simulated patients reported reductions in the inappropriate dispensing of medications at two of the three study sites.

Two studies investigated drug-related problems, one in the elderly [31] and the other in kidney disease patients [41]. The outcomes were measured using patient reports and pharmacists’ documentation. The interventions had a mixed impact. For example, both studies demonstrated that the pharmacists in the intervention groups interacted more with the patients and intervened more frequently to manage drug-related problems, with no impact on the number of refusals to dispense a medication, patients’ knowledge about the drug, adherence, or drug therapy problems.

Two studies examined treatment recommendations. One study used simulated patient visits to measure the impact of educational meeting and educational materials on the number of pharmacist-facilitated asthma plans and on pharmacists’ general communication skills using the Global Rating Scale [27]. The other study measured the impact of an electronic decision-support prompt to remind pharmacists to discuss the suitability of aspirin therapy with eligible diabetes patients [35]. Both interventions resulted in positive changes in outcome measures.

One study reported on the improvement in pharmacists’ ability to assess and teach correct inhaler technique skills with educational meetings, educational materials, audits, and feedback [22].

Based on the summary above, five studies reported a mixed impact, as some outcomes were favourably changed by the intervention and some outcomes were not changed. The unclear risk of bias in the majority of the studies provides little help in explaining the differences in reported findings among studies. Additionally, we did not observe any differences in the outcomes and the methods used to measure outcomes that could explain variations in the findings. As many of the studies did not perform sample size calculation, one explanation could be that the sample sizes were inadequate for detecting a difference between the intervention and control groups. Interestingly, one study was conducted in two settings using the same interventions, but the implementation of the interventions could explain the differences in effectiveness. For instance, the site at which a negative effect of the educational intervention was found was a seminar for large group and a voluntary peer review [23], while the other site involved face-to-face educational intervention and compulsory peer review [24]. This example illustrates how modifications of any intervention during the implementation process might impact outcomes. Furthermore, the pharmacists’ attitudes and working conditions may have differed between the two settings. In another study about smoking cessation, the same educational intervention was delivered to pharmacists and physicians separately, and the outcomes were assessed by patient report 12 months after intervention [34]. An increase in helping patients to quit smoking was found in the physicians in the intervention group, but not in the control group. Among the pharmacists, there was no difference between the intervention and control groups. This example suggests that factors other than knowledge and competence might impact outcomes. Examples of these factors include community pharmacists’ attitudes towards counselling, work hours, and staffing.

## Discussion

This study provides a review of the literature on interventions for improving pharmacist-led counselling in the community setting. The findings of the included 17 studies suggest that educational meetings combined with outreach visits and feedback have a positive effect on community pharmacists’ counselling in the community pharmacy setting. This finding is consistent with previous reviews on interventions for changing healthcare professionals’ behaviour [39, 42–44]. Johnson and May [42] conducted a review of systematic review articles to establish the characteristics of successful behavioural change interventions in healthcare. These authors identified 67 reviews examining the following three main categories of interventions: persuasive interventions (e.g. diffuse persuasive strategies, such as marketing and mass media, or direct persuasion strategies, such as local consensus processes and local opinion leaders); educational and informational interventions (e.g. patient-mediated interventions, dissemination of educational materials, educational meetings, and educational outreach); and action and monitoring interventions (e.g. audits, feedback, and reminders). The authors concluded that interventions focusing on actions or education tended to have more positive effects on professional behaviour than those based on persuasion. Several Cochrane reviews have shown that educational meetings [39], outreach visits [43], and audits and feedback [44] can improve professional practices and patient healthcare outcomes; however, the effect is most likely to be small and to vary according to many factors, such as the complexity of the behaviour targeted by the intervention. A previous review identified 11 studies that reported that the use of active learning through training using role play, feedback, and reflection was important for enhancing the communication skills of community pharmacists during consultations regarding non-prescription medications [17]. Another review identified 21 studies that reported that the use of training delivered as either didactic or interactive sessions had the potential to modify community pharmacists’ behaviour during service delivery [15].

The studies included in the present review contained insufficient information on methodology to permit judgement of the risk of bias, which necessitates caution in interpreting the findings. The subjective nature of outcome measures and the unclear blinding of outcome assessors also lowered our confidence in the findings. Furthermore, the short-term follow-up period precluded firm conclusions regarding sustained changes in the outcomes resulting from the interventions.

### Strengths and limitations

The present study offers a different perspective on community pharmacists’ counselling than was provided in most previous studies. In contrast to previous reviews [2–4, 10, 13, 14], we focused on studies investigating interventions for improving community pharmacist-led patient counselling. We deliberately narrowed the focus of the review to those studies that attempted to measure the impact of the intervention on the pharmacists’ behaviour during counselling and in a community setting. Our detailed examination of the types of interventions and assessment of the quality of the included studies provides insight into the strength of the evidence from the included studies and a greater understanding of the gaps in the literature.

Some limitations must be considered. Certain relevant papers may not have been included in our review, such as studies that are not indexed in the searched databases, studies published in languages other than English, and unpublished studies (grey literature). We attempted to ensure that our search strategy was as comprehensive as possible; however, it is possible that some papers describing counselling used different keywords, and in such cases, these papers could not have been identified for this review. There is no single definition of counselling in community pharmacy. Consequently, subjective assessments of study eligibility were required, which might have introduced some bias in the inclusion of studies. We did not contact the original studies’ authors to clarify many unreported study characteristics.

### Implications for future studies

The studies included in this review varied in the quality of reporting of study methodology and intervention components. Future studies should report their methods and findings in a comprehensive and transparent manner and describe intervention components in sufficient detail to facilitate evaluation and replication by others. We recommend the use of reporting guidelines for describing research and interventions [45, 46].

Counselling is a complex process influenced by the pharmacists, the customer, and their interaction [12]. Many factors influence the content and extent of the counselling provided by community pharmacists, including the type of medications (prescription only versus non-prescription medicine), the type of prescription (new versus repeat), type of presentation (product requests, symptoms, or conditions), and time constraints [12, 41, 47]. Pharmacy users’ expectations of their visit (i.e. buying a product versus obtaining a professional service) can influence the attempts of the pharmacy staff to engage the users in dialogue about their medicine use [48]. Additionally, pharmacy users’ interactions with community pharmacists are influenced by their perceptions of the professional role of the community pharmacist [48]. Furthermore, pharmacy users’ awareness of the need for questioning and their willingness to answer questions [49] are important factors. For instance, users may perceive the questions pharmacists ask as an attempt to control their medicine use, may find the dialogue irrelevant, or may not understand the pharmacists’ motivation for asking questions [12]. The discrepancies between pharmacy users and pharmacists’ expectations regarding illness and medicine have also been suggested as a barrier to optimal counselling [12]. Therefore, interventions should not be restricted to improving the knowledge and communication skills of the pharmacists. Counselling practices can be negatively influenced by the pharmacist’s workload, the pharmacy layout, lack of access to the patient’s health information, and the patient’s expectations of community pharmacists. Promising interventions that could be explored in future studies include delivery arrangements, such as staffing models; the use of information and communication technology; and governance arrangements, such as professional competence, training, and licensing. Future reviews on counselling in community pharmacy should synthesise evidence on interventions that target patients as part of the counselling process.

To identify effective behavioural change interventions, it is important to characterise the interventions [50]. The characterisation of interventions involves matching all possible intervention types to the behavioural target, the target population, the context in which the intervention is delivered, the underlying behavioural model, and the influencing factors [50]. Only six studies indicated the theoretical framework that was used to develop the interventions. We suggest that future studies characterise the interventions by describing the constructs of the framework used and how the constructs were integrated into the design of the intervention and the outcome measurement. Several frameworks for characterising behavioural change interventions exist. The behaviour change wheel is one of the few frameworks that meet all usefulness criteria of comprehensiveness, coherence, and a clear link to an overarching model of behaviour [50].

Future studies may consider examining individual interventions as the use of multifaceted interventions did not allow a clear understanding of the effectiveness of individual interventions. Previous reviews showed no compelling evidence that multifaceted interventions are more effective than single-component interventions in changing healthcare professionals’ behaviour [39, 51]. Comparisons of different types of interventions are also recommended.

We observed that the outcome measures in the identified studies were mainly subjective and focused on the quantity rather than the quality of counselling. There is a lack of reliable criteria or instruments for assessing the appropriateness of patient counselling in pharmacy practice. The variability in the outcome measures used among the studies further complicated the assessment of the interventions’ effectiveness. Researchers have attempted to describe the criteria used to assess the appropriateness of patient counselling in the community setting [52, 53]; however, more studies are needed to refine the criteria and establish a reliable instrument that is applicable for use in interventional studies.

Most of the reviewed studies documented changes in outcomes using simulated patient visits, a method that is commonly used in the pharmacy literature to develop and assess communication skills [40, 54]. Future studies should adhere to recommendations to improve the quality and validity of simulated patient visits, such as the use of standard data collection tools and audiotaping, if possible [54].

In the identified papers, we also observed inadequate discussion of the contextual circumstances and factors that can influence the delivery, implementation, and sustainability of the interventions. Factors that are crucial for successful implementation include costs, acceptability, and organisational changes. Future studies should elaborate on intervention implementation and sustainability issues to improve counselling by community pharmacists. For instance, frameworks have been proposed for assessing the acceptability of healthcare interventions during the development, piloting and feasibility, outcome and process evaluation, and implementation phases [55]. Future studies should utilise such frameworks to assess acceptability and facilitate the successful implementation of the interventions.

In this review, we focused on the effectiveness of interventions targeting counselling in the community pharmacy setting. Future reviews should identify evidence of the cost-effectiveness of these interventions. As the development and implementation of an intervention require a substantial resource commitment, it is important to have evidence on cost-effectiveness to aid decisions regarding resource allocation. This area has been neglected by researchers in this field [56].

## Conclusion

The included studies showed that educational meetings combined with educational materials, outreach visits, and feedback can improve pharmacists’ counselling in community settings. However, the unclear risk of bias and poor quality of reporting of intervention components necessitate caution in interpreting the findings.

## Additional files

Additional file 1: PRISMA checklist. (DOC 58 kb)

Additional file 2: Reasons for exclusion. (DOCX 24 kb)

Additional file 3: Summary of interventions and main findings of included studies. (DOCX 35 kb)

## Abbreviations

EPOC: The Cochrane Effective Practice and Organisation of Care
