# Supplementary material for: Interventions for improving pharmacist-led patient counselling in the community setting: a systematic review
Source: Syst Rev. 2018 May 2;7:71. doi: 10.1186/s13643-018-0727-4 (PMC5932789; doi:10.1186/s13643-018-0727-4)
Supplement: Supplementary file 2 — Reasons for exclusion. (DOCX 24 kb) [file 13643_2018_727_MOESM2_ESM.docx]

Additional file 2 Reasons for exclusion

| Study | Reason for exclusion |
| --- | --- |
| 1. Adepu et al. Indian J Pharm Sci. 2010;72(5):557-563 | Outcome measurement: did not measure the impact of intervention on counselling. Questionnaire of attitude, or theoretical knowledge |
| 1. [Airaksinen](https://www.ncbi.nlm.nih.gov/pubmed/?term=Airaksinen%20M%5BAuthor%5D&cauthor=true&cauthor_uid=9520965) (1999). [Med Care.](https://www.ncbi.nlm.nih.gov/pubmed/9520965) 1998 Mar;36(3):422-7. | Design: Not RCT, Not CCT, not CBA, not ITS. Not comparative (v) |
| 1. Ax et al. J Clin Pharm Ther. 2010;35(4):439–51. | Outcome measurement: did not measure the impact of intervention on counselling |
| 1. Ekedahl etal. J Clin Pharm Ther. 1996;21:95–99. | Design: Not RCT, Not CCT, not CBA, not ITS. uncontrolled before and after study |
| 1. [Beauchesne](http://journals.sagepub.com/author/Beauchesne%2C+Marie-France) et al. Journal of Pharmacy Technology 23(2): 67-74 | Patients discharged from hospital, not community setting although the services provided via community pharmacists. |
| 1. [Benrimoj](https://www.ncbi.nlm.nih.gov/pubmed/?term=Benrimoj%20SI%5BAuthor%5D&cauthor=true&cauthor_uid=19048386) et al. [Pharm World Sci.](https://www.ncbi.nlm.nih.gov/pubmed/19048386) 2009 Apr;31(2):230-7. | Design: Not RCT, Not CCT, not CBA, not ITS. Implementation non controlled study |
| 1. [Benrimoj](https://www.ncbi.nlm.nih.gov/pubmed/?term=Benrimoj%20SI%5BAuthor%5D&cauthor=true&cauthor_uid=19048386) et al. *International Journal of Pharmacy Practice* 2003; 11: 71–80 | The authors stated that Patient counselling was not considered as an intervention |
| 1. Bertsche etal. Int J Clin Pharm (2012) 34:17–22 | Design: Not RCT, Not CCT, not CBA, not ITS. Not comparative |
| 1. Bock et al. Nicotine & Tobacco Research.2010; 12:*217–225* | Design: Not RCT, Not CCT, not CBA, not ITS. Patients were randmoised not pharmacists. The intervention did not target pharmacists |
| 1. Barnett. Research in Social and Administrative Pharmacy 9 (2013) 27–36 | Participants: Targeting patients |
| 1. Crockett et al. *Aust. J. Rural Health* (2009) 17, 236–243 | Outcome measurement: did not measure the impact of intervention on counselling. Questionnaire of perception and theoretical knowledge |
| 1. [Curtain](https://www.researchgate.net/profile/Colin_Curtain) et al. British Journal of Clinical Pharmacology 71(5):780-4 | Outcome measurement: did not measure the impact of intervention on counselling. Impact on process only. |
| 1. De Vera et al. Trials 2014, 15:488 | Design: Protocol. Outcome measurement: did not measure the impact of intervention on counselling |
| 1. Geurts. Patient Education and Counseling 78 (2010) 85–90 | Participants: Targeting patients |
| 1. Hudmon etal . *J Am Pharm Assoc.* 2014;54:42–44. | Design: Not RCT, Not CCT, not CBA, not ITS. Also Outcome was pharmacists opinion not change in counselling practice |
| 1. Jackevicius et al. *Aust. J. Rural Health* (2009) 17, 236–243 | Outcome measurement: did not measure the impact of intervention on counselling. Questionnaire of knowledge |
| 1. Javadi et al. Evaluation & the Health Professions 2015, Vol. 38(3) 404-418 | Outcome measurement: did not measure the impact of intervention on counselling. measure pharmacists’ satisfaction, attitude, or theoretical knowledge after the intervention |
| 1. Jaffray et al. *International Journal of Pharmacy Practice* 2014; 22: 4–12 | Outcome measurement: did not measure the impact of intervention on counselling |
| 1. [Kradjan](https://www.ncbi.nlm.nih.gov/pubmed/?term=Kradjan%20WA%5BAuthor%5D&cauthor=true&cauthor_uid=10533347) et al. [J Am Pharm Assoc (Wash).](https://www.ncbi.nlm.nih.gov/pubmed/10533347) 1999 Sep-Oct;39(5):658-66 | Outcome measurement: did not measure the impact of intervention on counselling(patients satisfaction) |
| 1. Kristina et al. *Asian Pac J Cancer Prev,* 16 (8), 3319-3323 | Design: Not RCT, Not CCT, not CBA, not ITS. Also the only Outcome measuring counselling was measured after intervention and not before and after. |
| 1. Leemans. *Pharm World Sci 1998;20(6): 238-247* | Design: Not RCT, Not CCT, not CBA, not ITS. |
| 1. Legrand et al. Int J Clin Pharm (2012) 34:633–643 | Outcome measurement: did not measure the impact of intervention on counselling. Questionnaire measure pharmacists’ satisfaction, attitude, or theoretical knowledge after the intervention and intervention acceptablility |
| 1. Martin et al. Patient Education and Counseling 83 (2011) 319–324 | Design: Not RCT, Not CCT, not CBA, not ITS. Uncontrolled before and after study |
| 1. Martin et al. *J Am Pharm Assoc (2003)*. 2010 ; 50(1): 9–16 | Design: Not RCT, Not CCT, not CBA, not ITS. Uncontrolled before and after study |
| 1. McConnell et al. *Ann Pharmacother* 2010;44:1585-95. | Outcome measurement: did not measure the impact of intervention on counselling. Questionnaire of perceptions of their pharmacy practices |
| 1. McLean. Patient Education and Counseling 78 (2010) 85–90 | Design:Not intervention. Impact of providing pharmaceutical care |
| 1. Mestrovic. American Journal of Pharmaceutical Education 2012; 76 (2) Article 23. | Design: Not RCT, Not CCT, not CBA, not ITS. Uncontrolled before and after study |
| 1. Minhetal. Tropical Medicine and International. 2013, 18(4): 426–434 | Design: Not RCT, Not CCT, not CBA, not ITS. No control |
| 1. Minh et al. PLoS ONE 8(10): e74882. | Design: Not RCT, Not CCT, not CBA, not ITS. No control |
| 1. [Naunton](https://www.researchgate.net/profile/Mark_Naunton) et al. The Journal of Rheumatology 31(3):550-556 | Outcome measurement: did not measure the impact of intervention on counselling. |
| 1. Ngwerume 2015. Int J Pharm Pract. 23(2):102-10. | Outcome measurement: did not measure the impact of intervention on counselling (participants satisfaction) |
| 1. Obreli-Neto 2016. PLoS ONE 8(12): e79875 | Design: Not RCT, Not CCT, not CBA, not ITS. (participants questionnaire) |
| 1. Pick et al. Aids Care.1996;8(1):55-69. | Outcome measurement: did not measure the impact of intervention on counselling |
| 1. Raisch et al. Am J Health Syst Pharm. 1998;55(12):1274. | Outcome measurement: did not measure the impact of intervention on counselling. Process outcomes |
| 1. Simansalam etal. American Journal of Pharmaceutical Education 2015; 79 (5) Article 71. | Participant: Pharmacy students; Outcome measurement: did not measure the impact of intervention on counselling. |
| 1. Sarayani et al 2012. J Contin Educ Health Prof. 32(3):163-73. | Outcome measurement: did not measure the impact of intervention on counselling. Questionnaire of attitude, or theoretical knowledge |
| 1. Schickedanz 2013. Journal of General Internal Medicine .28(7):914-920] | Design: Not RCT, Not CCT, not CBA, not ITS. Training of multidisciplinary group |
| 1. Thoms et al . Patient Education and Counseling. 2016; 99: 132–138 | Design:Not comparative; Outcome measurement: did not measure the impact of intervention on counselling. Pharmacists confidence |
| 1. Van et al. BMC Health Services Research 2011; 11:313 | Outcome measurement: did not measure the impact of intervention on counselling |
| 1. Watson etal. Family Practice 2009; 26: 532–542. | Outcome measurement: to assess the acceptability of simulated patient  (SP) visits combined with feedback. |
| 1. Zillich et al. Research in Social and Administrative Pharmacy 9 (2013) 27–36 | Design:Methods and baseline findings only |
