# Supplementary material for: Interventions for improving pharmacist-led patient counselling in the community setting: a systematic review
Source: Syst Rev. 2018 May 2;7:71. doi: 10.1186/s13643-018-0727-4 (PMC5932789; doi:10.1186/s13643-018-0727-4)
Supplement: Supplementary file 3 — Summary of interventions and main findings of included studies. (DOCX 35 kb) [file 13643_2018_727_MOESM3_ESM.docx]

**Additional file 3 Summary of interventions and main findings of included studies**

| **Study (year),** | **Intervention** | **Main findings** |
| --- | --- | --- |
| Basheti (2009) | All received workshop covering basic asthma management, inhaled medications.  IG: a 3 hours training on how to assess and educate patients on inhaler technique+ how to teach peak flow meters (PFM) technique to patients + data form completion+ educational materials(included patient-centered educational tools for inhaler.technique education, including checklists for assessing technique and novel ‘‘inhaler technique labels’’)  CG: a 2 hours training on how to teach PFM technique to patients + data form completion.  Pharmacists were assessed and corrected on their inhaler technique if they performed any of the steps incorrectly by a researcher in 3 assessment visits. | Pharmacists in the IG had higher inhaler technique scores (out of 9) than pharmacists in the CG. Post training: Turbuhaler: IG, 8.7 +/- 0.9 vs. CG, 6.3 +/- 1.9; Diskus: IG, 8.6 +/- 0.8 vs. CG, 6.0 +/- 1.9; P = 0.002).  2 years after training Turbuhaler: IG, 8.7 1 0.9 vs. CG, 6.3 1 1.9; Diskus: IG, 8.6 6 0.8 vs. CG, 6.0 6 1.9; P = 0.002. |
| Chalker (2005) | Vietnam (Hanoi): Each pharmacy received two 45-min educational visits by a pair of senior researchers using both written and verbal information, one visit on dealing with requests for short course of antibiotics and the other on using oral steroids. Each session included the role of pharmacies in primary health care, information regarding good pharmacy practice, and information regarding ‘‘questions, advice, and treatment” strategy to improve case management + enforcement of regulations with local inspectors outreach visits+ compulsory peer review.  Thailand (Bangkok): a two days educational seminar for pharmacy owners and technicians on case management and rational use of drugs and a 2-hours face-to-face seminar delivered twice (one for antibiotic and one for steroids) for pharmacists who couldn’t attend the 2-days seminar+ enforcement of regulations with local inspectors outreach visits+ voluntary peer review. | Hanoi: significant reduction in the dispensing of illegal steroids dispensing in IG vs CG (17% vs. 57%, P<0.0001) and low dose antibiotics (71% vs. 95%, P0.0125), fewer dispensers asking no questions and giving no advice (11% vs. 30%, P0.0014 for steroids and 51% vs. 81%, P0.0028 for antibiotics).  Bangkok: insignificant reduction in illegally dispensing steroids of IG vs CG (56% vs. 74%) and antibiotic (88% vs 92%), and insignificant fewer dispensers asking no questions and giving no advice (72% vs 72% steroids and 72% vs. 81% antibiotics). |
| Chuc (2002) | Each pharmacy received two 45-min face-to-face educational visit by a pair of senior researchers using both written and verbal information on pharmacy treatment guidelines, and Question, Advice, and Treatment strategy were introduced (One for respiratory tract infection and requests for a short course of antibiotics; one for sexually transmitted disease and requests for oral steroids) + enforcement of regulations with local inspectors outreach visits+ peer review. | For ARI, antibiotic dispensing decreased (P <.02) and questions regarding breathing increased (P <.01). For STD, advice to go to the doctor and dispensing the correct syndromic treatment increased (P <.01). Dispensing of prednisolone and cefalexin decreased (P <.01) and prescription requests increased (P <.01). |
| de Almeida Neto (2000)  Australia | A pharmacy protocol in which purchasers of non-prescription analgesics are asked to review their use of the medication was taught to pharmacists during a 3 hours workshop. This protocol is based on the Stages of Change model and on the principles of motivational interviewing. For example, pharmacists prompted consumers to talk about their medication use and assesses the consumer’s readiness to change. A researcher provide pharmacists with positive feedback on positive features of their performance and corrective feedback that addressed how pharmacists could improve performance. | IG were more accurate at identifying people who misused the medication (*P*<0.001), more likely to use open-ended questions (*P*<0.001), assess readiness to change problematic use (*P*<0.001), and to deliver a brief intervention that was tailored to the person’s commitment to alter his/her usage (*P* <0.001). |
| de Almeida Neto (2000)  Australia | A pharmacy protocol in which purchasers of non-prescription analgesics are asked to review their use of the medication was taught to pharmacists during a 3 hours workshop. This protocol is based on the Stages of Change model and on the principles of motivational interviewing. For example, pharmacists prompted consumers to talk about their medication use and assesses the consumer’s readiness to change.  A researcher provide pharmacists with positive feedback emphasising segment of the protocol that were performed correctly and corrective feedback that addressed how pharmacists could improve performance. | IG were more accurate at identifying people who misused the medication (29% vs 13%), and to discuss use of alternative (32% vs 6%). |
| Dolovich (2007)  Canada | A one-day workshop. The workshop focused on therapeutic topics including knowledge of asthma and its drug therapies, the role of peak expiratory flow monitoring, and inhaler and device use, skill-building topics focused on how to introduce the concept of asthma self-management with patients, a structured interview process for assessing patients and how to develop a patient’s asthma action plan  Two follow-up telephone calls at 2 and 4 weeks post-intervention. A tool kit of resources for pharmacists was provided during the workshop.  The control group received the workshop after the primary outcome measurement. | Pharmacists in IG produced an appropriate plan in 44.8% of situations (117/261) compared with 29.3% (79/270) in the CG  [95% CI on difference, 7.4–23.8%] (P = 0.0004).  IG pharmacists exhibited better overall communication skills including  empathy, coherence, verbal skills, and nonverbal skills. |
| Garcia (1998)  Peru | An 8 hours training course on sexually transmitted disease (STD) recognition, management, and prevention counselling. A 1.5-2 hours session was offered on pharmacy site for those who could not attend the workshop. Manual for pharmacy worker and pamphlet for their client was also developed. | IG (n=172 visits) ) and CG (n=176 visits) did not differ except IG suggested more partner treatment (18% vs 8.5 %; p<0.01), abstinence during treatment (21% vs 13 %; p=0.04), counselling for genital ulcer (44% vs 15 % ; p=0.004) and pelvic inflammatory disease (56% vs 36 % ; p=0.04) |
| Garcia (2003)  Peru | A 90 minute interactive luncheon seminars on recognition  and management of four STD syndromes and STD/HIV prevention counselling+ Pocket-size folding cards and posters that summarized management of each STD syndrome, , manuals about recognition, management, and prevention of and counselling for STD, displays and lapel pins stating “I know about STD, ask me”, white coats with STD logo for pharmacy workers, listings of certified referral physicians and clinics+ monthly pharmacy visits by “prevention salespersons” who distributed materials for client that included “STD/HIV prevention packets” containing information, condoms, and cards given to patients for referral of their sex partners.  CG received training on diarrhoea management. | IG had significantly better adherence *(P≤*0.05) to training guidelines for 47 of the 48 the primary outcome measures at 1, 3, and 6 months (4 outcomes for each of 4 conditions assessed 3 times). This include substantially and significantly (*P*<0.05) higher rates of discerning and discussing each of the four syndromes as possibly representing STD; of adequate management offered for the syndrome;  and of recommendations for condom use (except for pelvic inflammatory disease, (*P* = 0.06) and for referral of partners for treatment. |
| Kimberlin (1993)  USA | Home study using a training manual+1 day workshop + copies of patient information and leaflets + compliance aids such as reminder alarm+ free access to Drug Information Services.  The theoretical foundation used to help pharmacists conceptualize patient problems with drug use and address these problems were the cognitive-behavioural and multi modal therapy. | The odds ratio pf IG patients reporting that the pharmacists discuss new medications versus not discussion new medications, asked about problems during refill, gave written information and gave advise were higher than the for the CG. This trend continued at 3 months follow up.  There was no difference between the two groups in patients’ knowledge about the drug, adherence or and drug therapy problems |
| Lalonde (2008)  Canada | ProFiL Program comprises a 3 hours training workshop; a communication-network program involving a list of chronic kidney disease patients medications as documented by the nephrologist, a clinical summary of their health problems and laboratory test results; and consultation service from hospital pharmacists. | A mean of 0.50 opinion per IG patient and 0.02 per CG patient were issued (adjusted 95% CI difference: 0.28–1.01opinion/patient).  The number of refusals to dispense a medication was low and similar in both groups before and during the study. |
| Mayer (1998)  USA | A 23-minutes videotape and accompanying materials+ written and verbal feedback on counselling performance+ incentives, skin cancer prevention brochures and prompts such as sunscreen samples and posters.  The video contained didactic information about skin cancer prevention, Ask, Advice and Assist model+ 6 scenes of pharmacist patient interactions. | Before intervention, 0% of the IG sites and 7.4% of the CG sites provided counselling (ns)  After intervention, 18 (66.7%) of the IG sites and 1 (3.7%) of the CG sites provided counselling (P<.001) |
| Patwardhan (2012)  USA | The CG received Quit line cards, an informational presentation about quit line services, and enrolment in Fax to Quit (FTQ), which is a free service. FTQ enabled pharmacies to proactively refer tobacco users to the quit line by faxing a signed consent form that allowed the quit line to directly call-back users to initiate cessation treatment. The CG received all that was provided to the CG plus a multimodal intervention that consisted of a 30-minutes on site training to conduct a brief tobacco cessation counselling by using the ask–advise–refer (AAR) approach; self-study script; a tobacco cessation poster, guidance on implementing AAR in pharmacy workflow, and a support visit by the author 1 week into AAR implementation to identify and resolve any challenges  faced in implementing AAR.  The intervention drew from the authors’ formative qualitative study, literature on smoking cessation, and constructs from the social cognitive theory. | The number of customers asked by pharmacists about tobacco use (P <0.001), number of tobacco users advised to quit (P< 0.01), and number of tobacco users enrolled in the Quit Line via FTQ (P = 0.001) and number quit line cards given (P<0.05) were higher in IG. |
| Prokhorov (2010) | Two hours of training demonstrating medications for cessations, practicing counselling through use of role-playing, and videos were used to demonstrate appropriate counselling interactions. Patient hand- outs in English and Spanish, and printed materials (i.e., posters and leaflets) were also provided.  IG received training on smoking cessation counselling  CG received training on skin cancer prevention counselling | For patients counselled by pharmacists, there were no significant differences between the IG and CG from baseline to 12 months and no difference between control and intervention for the pharmacists counselling activities for each of the 5 A’s counselling practice model: ask, advice, assess, assist, and arrange.  At 12 months post training, IG pharmacists’ perceived ability, confidence, and intention (ACI) to address counselling activity index increased from pre-training to post-training (4% to 21%; P < .04). In CG, no significant increase was detected (10% to 19%; P = .99). |
| Reeves (2007) | An electronic decision-support prompt identifying patients for a  targeted proactive clinical intervention was developed and implemented. Each time an oral antidiabetic agent was dispensed, a prompt was displayed reminding pharmacists to discuss the suitability of aspirin therapy in eligible patients with diabetes. This recommendation is supported by current clinical guidelines. Half intervention pharmacies allocated observer to remind the pharmacists to document, but not to prompt them to perform an intervention | The overall documented clinical intervention rate while the aspirin prompt was active was found to be 1.74 per 100 patients (95% CI 1.55, 1.93) in the IG versus 0.91 (0.77, 1.05) for the control (*P* < 0.001).  IG pharmacists recorded a total of 201 target interventions related to  aspirin therapy in diabetes( 2.55 interventions per 100 diabetic patients (95% CI 0.85, 4.24). No targeted interventions were recorded in the control group. The presence of observers to assist in documentation and to remind the pharmacists to document, but not to prompt them to perform an intervention increased the rate of recorded clinical interventions in the prompt arm of the trial. For the ‘observer arm’ of the trial, the rate was approximately four  times that of the non-observed arm (7.52 *vs.* 2.00 interventions  per 100 diabetic patients).  The effect of the prompt decreased over the study period and was not maintained after prompt deactivation. |
| Sigrist (2002) | Seven simulated patients visits at which requests were made for non-prescription medicines. Visit 1 and 2 were for collection of baseline data. Training was given after visit 2 and direct feedback to the pharmacy staff was given after visits 3 , 4, 5, 6  and 7. Training workshop (5 hours) incorporated roleplays, an explanation and demonstration of the theoretical models and discussion of the practical protocol and the pseudo-patient methodology.  The stages of change model and the health belief model were used to develop the practical protocol. | Total scores obtained at each visit time point post-training (visits 3 to 7) in IG pharmacies (98 interactions) significantly higher than those in the CG group (91 interactions). The score in IG visit 7 was lower than in previous visits. |
| Sinclair (1998) | A two-hour workshop based on the stage-of-change model including how to move smokers from the contemplation and preparation stages to the action and maintenance stages (case studies of pharmacy customers were used) and communication skills for negotiating change (how to briefly question and assess the customer’s stage of readiness and tailor their advice to the current stage of the customer). The workshop did not focus on smoking cessation products. | Intervention customers were more likely to discuss stopping smoking with the pharmacist (85%) compared to (62%) of the control (P<0.001) and also rated discussion more highly (34% vs 16%, P=0.048).  Smoking cessation outcomes as reported by patients were higher in IG compared to CG but was not statistically significant. |
| Watson (2002) | The interventions included dissemination of an evidence-based guideline for OTC management of vulvovaginal candidiasis (thrush) by postal dissemination (control,n=15), educational outreach visit (n=15) , continuing professional education session (n=15), or educational and outreach visit (n=15). | There were no significant differences in the proportion of appropriate outcomes following educational outreach [odds ratio (OR) = 1.1; 95% confidence interval (CI) 0.52 to 2.45] or continuing professional education (OR = 0.88; 95% CI 0.41 to 1.91). |
